# Explosion in the Size of the Elderly Population

**Published:** 1990-09

**Authors:** Vernon Coleman


### Explosion in the Size of the Elderly Population

**Vernon Coleman MB ChB**

In the next thirty years age will have a far more divisive effect on our society than race, sex or class have ever had.

In the decade between 1971 and 1981 the total population of Britain increased by less than one percent. In that same period the pensionable population rose by 10%. One person in five in Britain is already a pensioner. By the year 2020 a third of the British population will be pensioners.

Several things make this explosion in the size of the elderly population particularly dangerous.

First, there is the fact that among older populations there is inevitably a higher proportion of disabled and dependent individuals. The incidence of chronic disease rises rapidly among older age groups. Among 16 to 44 year olds 20% of the population suffer from chronic illness. Among 45 to 64 year olds 40% of the population suffer from chronic illness. Among 65 to 74 year olds 50% of the population suffer from chronic illness. Among 65 to 74 year olds 50% of the population suffer from chronic illness. And among people aged 75 or over 65% of the population will suffer from chronic, long term illness.

The size of the potential problem can be estimated from the fact that in an article in the British Medical Journal last year Robert Anderson, Senior Research Officer at the Institute for Social Studies in Medical Care estimated that there are already more than one and a quarter million people in Britain caring for disabled or elderly people living in the community.

To illustrate the sort of impact this increase in illness is likely to have consider blindness. Among the general population the incidence of blindness runs at about 200 per 100,000 people. But among the over 75s the incidence of blindness approaches 8,000 per 100,000 individuals.

Or consider strokes. Already half the beds in National Health Service hospitals are occupied by patients suffering from some sort of stroke. Stroke patients generally need to stay in hospital for long periods of time and they need intensive nursing care.

Or consider diabetes. Since diabetes is a hereditary disease the incidence of this problem is increasing rapidly. The increase in the number of elderly people alive in our society merely exacerbates the problem. Experts now estimate that the incidence of diabetes is doubling every decade. In 1984 just 2% of the population was diabetic. If the increase continues at the same rate then by the year 2020 (less than half a lifetime away) one in four people will be diabetic.

Nor do we only have to face an increase in the incidence of physical disease. There is also ample evidence to show that mental disease is on the increase too. In the last decade for which figures are available the number of patients with mental illness needing to be admitted to hospital rose by nearly 10%. Today between 10% and 15% of the population will, at some stage in their lives, suffer from mental illness severe enough to warrant their admission to a mental hospital. Once again it is the increase in the size of our elderly population which is at least partly responsible.

One side effect of all this will be that more and more of our hospital beds will be blocked and unavailable for emergencies. Dr John Loraine, Senior Lecturer in the Department of Community Medicine and Director of the Centre for Human Ecology at the University of Edinburgh has estimated that by 1992 94% of all non maternity beds for women and 75% of all beds for men in non psychiatric hospitals could be occupied by patients aged 65 or over.

All this will inevitably mean that waiting lists for non urgent surgery will get longer and longer and the number of people in our community suffering from disabling problems (such as arthritic hips) will grow even faster.

To all this we must also add the fact that in Britain the average cost of health care for individuals aged between 65 and 74 is $2\frac{1}{2}$ times as much as it is for individuals aged between 15 and 64. For people over the age of 75 the cost of providing health care rises to 7 times the cost of looking after patients under 64. So, the steady increase in our elderly population will mean that the NHS will become increasingly short of funds.

The second reason why the explosion in the size of our elderly population is dangerous is that the number of young people seems to be falling.

The most obvious reason for this is that we have become very efficient at persuading young couples to have fewer children. But that isn't the only reason.

The imbalance between the size of our elderly population and the size of our young population is also made worse by the fact that whereas people in their 60s, 70s and 80s seem to be living longer and longer, death rates among people in their 20s and 30s seem to be increasing. The number of young people (particularly men) dying of heart disease is showing no signs of falling. And the number of young people committing suicide is rising.

The result of all this is that a smaller and smaller working population will have to support a larger and larger dependent population.

And that takes us nearly into the third reason why the explosion in the size of our over 60 population is likely to produce real problems: money.

Most workers who are currently paying pension contributions assume that the money they are paying will be invested and repaid to them when they reach pensionable age. But that is not the case, of course. The pension contributions paid by today's workers are used to pay the pensions of yesterday's workers—today's pensioners.

The pensions that today's workers will receive when they retire will be paid by the regular contributions made by tomorrow's workers.

But the working population is getting much smaller. And the retired population is getting much bigger.

You don't have to be a genius to see the disaster we're heading for.

By the end of the century the size of our elderly population will have begun to concern politicians. By then it will be too late. This is a problem we should be worrying about now. It is, without a doubt, the biggest problem our society has to deal with.

